# Les tumeurs du grêle: à propos de 27 cas

**DOI:** 10.11604/pamj.2018.30.13.5407

**Published:** 2018-05-08

**Authors:** Ihsane Mellouki, Khaoula Jellali, Adil Ibrahimi

**Affiliations:** 1 Service d’Hépato-Gastroentérologie C4, CHU Hassan II, Faculté de Médecine et de Pharmacie, Université Sidi Mohammed Ben Abdallah, Sidi Hrazem, Fès, Maroc

**Keywords:** Intestin grêle, cancer, aspect endoscopique, types histologiques, Small bowel, cancer, endoscopic appearance, histologic types

## Abstract

Les tumeurs gréliques représentent 5% de l'ensemble des tumeurs du tractus digestif. Leur pronostic reste sombre vu le retard diagnostic dû au manque de spécificité des manifestations cliniques et à la difficulté d'exploration bien que d'important progrès ont été fait sur le plan radiologique et endoscopique. Le but de ce travail est de préciser les aspects endoscopiques et histologiques des tumeurs gréliques. Il s’agit d’une étude rétrospective descriptive menée au Service d'Hépato-gastro-entérologie du CHU Hassan II de Fès sur une période de 11 ans (2002-2012). Nous avons analysé les aspects épidémiologiques, cliniques, endoscopiques et histologiques. 27 cas avaient été colligés. L'âge moyen était de 48 ans [21-80ans] avec une prédominance masculine (H/F: 2,4). La tumeur avait été révélée par des mélénas chez 55%, une masse abdominale chez 15% et un syndrome de Koenig ou syndrome occlusif chez 11%. Un antécédent de maladie cœliaque avait été retrouvé dans un cas. L’entéroscanner avait pu préciser le siège de la tumeur chez 18 cas. L’entéroscopie poussée pour les tumeurs proximales et l’entéroscopie double ballonnets pour les tumeurs à localisation distale avaient été réalisées chez 8 patients (30%), l'aspect endoscopique avait révélé une lésion ulcérée chez 37,5%, ulcérée sténosante chez 50% et ulcéro-bourgeonnante sténosante chez 25%. Dans 18 cas, c’est la chirurgie avec étude anatomopathologique de la pièce opératoire qui avait permis de poser le diagnostic. Le type histologique été dominé par les tumeurs stromales 14 cas (51%), suivi des adénocarcinomes 5 cas (18,5%), 4 cas (15%) de LMNH de type B, un cas de carcinome neuroendocrine jéjunal. Les tumeurs gréliques sont rares mais de pronostic sombre, les méthodes actuelles d'imageries (Entéroscanner et Entéro IRM) couplées aux examens endoscopiques, précisément l’entéroscopie double ballonnet, devraient permettre un diagnostic plus précoce et une diminution de la mortalité.

## Abstract (en)

Tumors of the small bowel account for 5% of all tumors of the gastrointestinal tract. Although important progress has been made from a radiological and endoscopic point of view, they have a poor prognosis due to delayed diagnosis. This occurs because of the absence of clinical manifestations and of the difficulties of exploration. This study aimed to highlight the endoscopic and histologic features of tumors of the small bowel. We conducted a retrospective descriptive study in the Department of Hepato-Gastroenterology at the University hospital Hassan II, Fez, over a period of 11 years (2002-2012). We analyzed the epidemiological, clinical, endoscopic and histologic features of tumors of the small bowel. Data were collected from the medical records of 27 patients. The average age was 48 years [21-80 years] with a male predominance (H/F:2.4). Melenas led to the discovery of tumor in 55% of cases, abdominal mass in 15% of cases and Koenig syndrome or occlusive syndrome in 11% of cases. One patient had a history of celiac disease. CT enteroclysis showed the seat of the tumor in 18 cases. Push enteroscopy for detection of proximal tumors and double-balloon enteroscopy for detection of distal tumor were performed in 8 patients (30%). Endoscopy showed ulcerated lesion in 37.5% of cases, ulcerated stenotic lesion in 50% of cases and ulcerobudding stenotic lesion in 25% of cases. Surgery with anatomopathological examination of the surgical specimen allowed the diagnosis in 18 cases. The histologic type was dominated by stromal tumors in 14 cases (51%), followed by adenocarcinomas in 5 cases (18.5%), B-cell non-Hodgkin lymphoma in 4 cases (15%), neuroendocrine carcinoma of the jejunum in 1 case. Tumors of the small bowel are rare but they are characterized by a poor prognosis. Current imaging techniques (CT enteroclysis and MR enterography) coupled with endoscopic examinations, in particular double-balloon enteroscopy, allow earlier diagnosis and mortality reduction.

## Introduction

Les tumeurs malignes primitives de l’intestin grêle (TMPIG) sont des tumeurs rares. Elles représentent 1 à 5% de toutes les tumeurs du tube digestif bien que l’intestin grêle représente 75% de la longueur totale et plus de 90% de la surface muqueuse du tractus digestif. La multiplicité des types histologiques associés à la rareté de ces tumeurs et l’absence d’essais randomisés prospectives élucidant les meilleures options diagnostiques et thérapeutiques rendent difficile d’établir des statistiques valables. L’intestin grêle est considéré comme une zone cliniquement silencieuse et il en résulte un retard diagnostic et donc un traitement non optimal et un pronostic sévère. Nous rapportant les caractéristiques épidémiologiques, diagnostiques des patients atteints de tumeurs de l’intestin grêle ainsi que leurs aspects endoscopiques et histologiques colligés au sein au service d'hépato-gastro-entérologie du CHU Hassan II de Fès.

## Méthodes

Il s’agit d’une étude rétrospective descriptive des dossiers cliniques de 27 patients ayant le diagnostic de TMPIG admis dans notre formation sur une période de 11 ans s'étalant de 2002 à 2012. Tous les patients ayant une confirmation histologique d’une tumeur maligne du duodénum jusqu’à l’iléon terminal ont été inclus dans l’étude. Le diagnostic de TMPIG a été posé sur une étude anatomopathologique soit de la pièce de résection chirurgicale intestinale ou sur une biopsie tumorale. Pour chaque dossier, les paramètres suivants ont été étudiés: l’âge, le sexe, les données cliniques, para-cliniques ainsi que les aspects endoscopiques, histologiques.

## Résultats

Nous avons colligés 27 cas de tumeurs de l’intestin grêle en 11 ans, ce qui représente 4,2 % des 464 tumeurs digestives colligées durant la période. Sa fréquence chez le sujet jeune de moins de 45 ans était de 48 % (N: 13). L’âge des patients atteints de tumeur de l’intestin grêle, au moment du diagnostic variait de 21 à 80 ans avec une moyenne de 48 ans. Dix-neuf malades étaient de sexe masculin et 8 de sexe féminin, avec un Sexe ratio H/F: 2,4. Dans les antécédents des patients, 2 cas de maladie cœliaque associée. Concernant le mode de déclaration, la tumeur était révélée par des mélénas avec des douleurs abdominales dans plus de la moitié des cas pour chacun 55,5% (N: 15), suivie des vomissements chroniques dans 22% des cas (N: 6), ensuite venait le syndrome tumoral avec 4 masse abdominale révélant la maladie (15%). Nous avons eu 3 cas de syndrome de Koenig et 3 cas de syndrome occlusif soit 11% chacun. Il est à noter que l’amaigrissement représentait la plainte la plus fréquente retrouvée chez presque tous les patients 81,5% (N: 22). Une échographie abdominale était réalisée chez 19 malades (70%): un épaississement pariétal grêlique était retrouvée dans 8 cas (42%), une masse tissulaire chez 6 malades (31 %), avec dans 5 cas (26 %) des adénopathies rétro-péritonéales et mésentériques et une ascite dans 10, 5% des cas (N: 2). L'entéro-scanner réalisé chez 24 cas (89%) a montré un épaississement pariétal grêlique localisé dans 67% des cas (N: 16) et étagée dans 8% des cas (N: 2). Un aspect de sténose localisée dans 4 cas (17%), une masse tissulaire au dépend du grêle 2 cas (8%), 2 cas d’invagination intestinale (8%), 1 cas de mésentérite rétractile.

**Figure 1:**
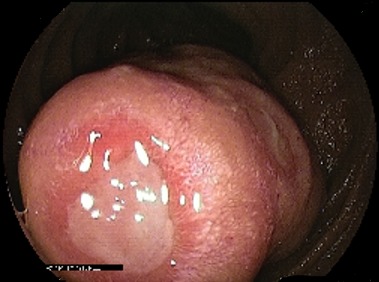
Processus ulcéro-bourgeonant - tumeur stromale jéjunale

**Figure 2:**
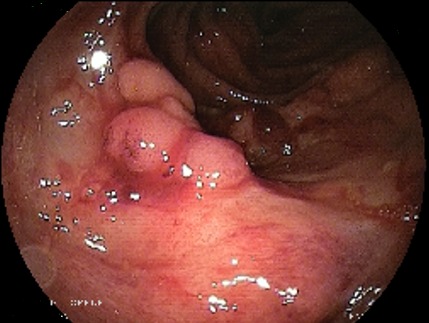
Aspect ulcéré - lymphome du grêle

**Figure 3:**
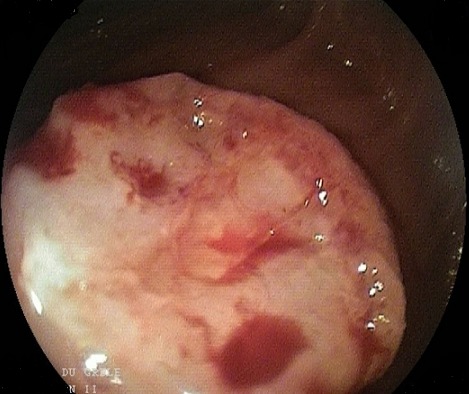
Tumeur ulcéro-bourgeonante sténosante - adénocarcinome jejunale

**Figure 4:**
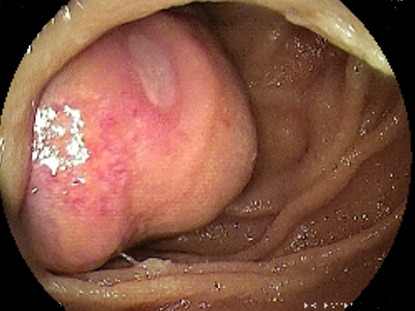
Aspect ulcéré - adénocarcinome duodénal

L’entéroscopie poussée pour les tumeurs proximales et l’entéroscopie double ballonnets pour les tumeurs à localisation distale étaient réalisées chez 8 patients (30%), l'aspect endoscopique avait révélé une lésion ulcérée chez 37,5% (N: 3), ulcérée sténosante chez 37,5% (N: 3) et ulcéro-bourgeonnante sténosante dans 2 cas (25%), (Figure 1, Figure 2, Figure 3, Figure 4). La biopsie avec étude anatomopathologique avait permis de poser le diagnostic dans 87,5 des cas (N: 7). Dans 18 cas, c’est la chirurgie avec étude anatomopathologique de la pièce opératoire qui avait permis de préciser le type histologique. Les résultats globaux des examens anatomopathologiques des tumeurs du grêle révèlent une nette prédominance des tumeurs stromales qui dépassent la moitié des cas (51%), surtout de haut grade dans 29 % des cas, suivi des adénocarcinomes (18,5%) avec dans 2 cas un ATCD de maladie cœliaque associée et des LMNH (15%), avec 1 cas de carcinome neuroendocrine. Les tumeurs du grêle siégeaient avec prédilection dans 63 % des cas au niveau jéjunal (N: 17).

## Discussion

Les tumeurs malignes de l'intestin grêle sont rares partout dans le monde [1], avec une incidence globale de moins de 1 pour 100.000 habitants [2]. Les cancers de l'intestin grêle ne représentent que 0,42% du taux totales des cancers et 2,3% des cancers digestifs aux États-Unis [3], tandis qu'au Canada, sont taux est de 0,37% et 1,78% respectivement [4]. Dans notre série son taux est relativement plus élevé de 4,2% par rapport aux autres cancers digestifs, il rejoint celui retrouvé dans une série africaine 5,4% [5], mais reste toutefois faible par rapport aux autres localisations. Dans notre série, le cancer du grêle apparaît à un âge jeune, 48 ans en moyenne. Sa fréquence chez le sujet jeune de moins de 45 ans était de 48 %. Halima A et al. ont rapporté des résultats similaires avec un âge moyen de 46 ans [6]. Ces résultats sont nettement inférieur à ceux rapportés par de nombreux auteurs qui ont trouvé une moyenne d’âge située dans la sixième décennie [7]. L'incidence chez l’homme est considérée comme plus élevée que chez la femme pour la plupart des pays et cela pour tous type histologique [6-8]. Notre série rejoint ces données avec un Sexe ratio H/F: 2,4. Les patients suivi pour maladie cœliaque ont un risque élevé de cancer de l'intestin grêle type lymphome non hodgkiniens à cellules T et adénocarcinome [8,9]. En effet, le risque relatif de ce dernier est estimé à 60-80. Ces carcinomes sont le plus de siège jéjunal [8]. Dans notre série nous avons recensé 2 cas d’adénocarcinome de siège jéjunal chez deux patients ayant comme antécédent une maladie cœliaque.

Les manifestations cliniques ont tendance à être non spécifique. De ce fait, le diagnostic est posé tardivement, généralement au stade métastatique (70%) [7]. Les symptômes dépendent habituellement du site et de la taille de la tumeur. La plainte la plus fréquente (83%) est représentée par les douleurs abdominales et la perte de poids suivie par l'anémie et hémorragie digestive type méléna [7,10]. Dans notre série, la perte de poids était retrouvée chez tous nos patients (82%), les douleurs abdominales et les mélénas étaient les symptômes révélateurs les plus fréquents avec un taux de 55,5% pour chacun. Le tableau d’occlusion intestinale et/ou de sub-occlusion représente un mode de révélation fréquent cité par la plupart des auteurs. Abahssain H et al. rapporte un taux de 37% [6]. Ce taux peut varier de 16-65% selon les études [7], dans notre série il était de 22 %.

De nouvelles méthodes diagnostiques comme l'entéro-scanner, la vidéo-capsule, ou l'entéroscopie permettent actuellement une meilleure exploration des tumeurs de l’intestin grêle. L'entéro-scanner présente une sensibilité de l’ordre de 95% et une spécificité de 96% pour le diagnostic des tumeurs du grêle [11]. Dans notre série, il a permis de préciser le siège de la tumeur et de déterminer son bilan d'extension dans 75% des cas (N: 18). Le 2^ème^ moyen diagnostique qui est d’actualité récemment est l’entéroscopie poussée et l’entéroscopie double ballonnets. Selon une étude chinoise, ce moyen, généralement bien toléré, permettait en cas de bilan de saignement digestive inexpliqué de mettre en évidence la cause dans 75% des cas et dont 39% des tumeurs de l'intestin grêle [11]. Dans notre série elles étaient réalisées chez 8 patients (30%), l'aspect endoscopique avait révélé une lésion ulcérée chez 37,5% (N: 3), ulcérée sténosante chez 37,5% (N: 3) et ulcéro-bourgeonnante sténosante dans 2 cas (25%). Le diagnostic histologique a pu être posé par ce moyen dans 87,5 % des cas.

Dans la littérature, de nombreux auteurs rapportent que les adénocarcinomes sont les types histologiques les plus fréquents des cancers de l’intestin grêle suivis par les tumeurs carcinoïdes et les LMNH [5-8]. Les résultats de notre étude diffèrent et révèlent une nette prédominance des tumeurs stromales qui dépassent la moitié des cas (51%), surtout de haut grade dans 29% des cas, suivi des adénocarcinomes (18,5%) avec dans 2 cas un ATCD de maladie cœliaque associée, les LMNH (15%) viennent en 3^ème^ position et 1 cas de carcinome neuroendocrine (4%). Ces tumeurs siégeaient avec prédilection au niveau jéjunal quelque soit le type histologique, ce qui rejoint les données de la littérature [8, 11-13].

## Conclusion

Les tumeurs de l’intestin grêle restent un challenge diagnostique pour les cliniciens, cependant leur diagnostic peut être aidé par les nouveaux moyens radiologiques (Entéroscanner et EntéroIRM) couplées aux examens endoscopiques, précisément l’entéroscopie double ballonnet qui ont permis d’explorer cette zone du tube digestif qui était jusque là une zone sombre, assurant de ce fait un diagnostic plus précoce et une meilleure prise en charge.

### Etat des connaissances actuelles sur le sujet

- Les tumeurs du grêle sont des tumeurs rares (5% des cancers digestifs);
- Les adenocarcinomes et les tumeurs endocrines sont les plus fréquentes (40%);
- Pronostic sombre, diagnostic généralement tardive vu ses manifestations cliniques polymorphes.

### Contribution de notre étude à la connaissance

- Avec une fréquence de 4,2%, les tumeurs du grêle reste rares et ça rejoins les données de la littérature;
- Contrairement aux données de la littérature, dans notre étude les tumeurs stromales sont prédominant avec une fréquence de 51%.

## Conflits d’intérêts

Les auteurs ne déclarent aucun conflit d'intérêts.
